# Negative parental and offspring environmental effects of macroalgae on coral recruitment are linked with alterations in the coral larval microbiome

**DOI:** 10.1098/rsos.240187

**Published:** 2024-07-24

**Authors:** Chloé Pozas-Schacre, Hugo Bischoff, Camille Clerissi, Maggy M. Nugues

**Affiliations:** ^1^ PSL Université Paris: EPHE-UPVD-CNRS, UAR 3278 CRIOBE, Université de Perpignan, 66860 Perpignan, France; ^2^ PSL Université Paris: EPHE-UPVD-CNRS, UAR 3278 CRIOBE BP 1013, 98729 Papetoai, Mo'orea, French Polynesia; ^3^ Laboratoire d'Excellence CORAIL, Perpignan, France

**Keywords:** coral reefs, microbiome, coral–algal competition, macroalgae, coral recruitment, parental effects

## Abstract

The persistence of reef-building corals is threatened by macroalgal competitors leading to a major demographic bottleneck in coral recruitment. Whether parental effects exist under coral–algal competition and whether they influence offspring performance via microbiome alterations represent major gaps in our understanding of the mechanisms by which macroalgae may hinder coral recovery. We investigated the diversity, variability and composition of the microbiome of adults and larvae of the coral *Pocillopora acuta* and surrounding benthic substrate on algal-removed and algal-dominated bommies. We then assessed the relative influence of parental and offspring environmental effects on coral recruitment processes by reciprocally exposing coral larvae from two parental origins (algal-removed and algal-dominated bommies) to algal-removed and algal-dominated environmental conditions. Dense macroalgal assemblages impacted the microbiome composition of coral larvae. Larvae produced by parents from algal-dominated bommies were depleted in putative beneficial bacteria and enriched in opportunistic taxa. These larvae had a significantly lower survival compared to larvae from algal-removed bommies regardless of environmental conditions. In contrast, algal-induced parental and offspring environmental effects interacted to reduce the survival of coral recruits. Together our results demonstrate negative algal-induced parental and offspring environmental effects on coral recruitment that could be mediated by alterations in the offspring microbiome.

## 1. Introduction

Corals live with a striking diversity of microbial symbionts (Symbiodiniaceae, bacteria, archaea, viruses, fungi) that underpin host health through metabolic and defence functions [1]. To establish and maintain associations with microbes across life stages is therefore essential for the holobiont survival. Microbial symbionts can be acquired through the environment (i.e. horizontal transmission) [2], the parents (i.e. vertical transmission) [3] or a combination of both (i.e. mixed strategy) [4,5]. A dynamic microbiome structuring occurs throughout coral ontogeny, with juvenile corals harbouring a more diverse and variable microbiome than adult microbiomes [4,6]. This ‘winnowing process’ during early life stages of marine invertebrates has been suggested to enable the establishment of fine-tuned microbial associations for a given environment [7]. Consequently, characterizing how environmental factors shape the coral microbiome at different life stages is critical to identify key host–microbe associations and to assess their stability or flexibility under stressful conditions.

On coral reefs, the cumulative impact of human-driven stressors (e.g. warming ocean temperatures, overfishing, pollution) has resulted in extensive changes in benthic assemblages leading to phase shifts from coral to macroalgal dominance [8]. Once established, macroalgae can compromise the survival, growth, recruitment and fecundity of corals [9–12]. Microbially mediated coral–algal competition has been suggested as one of the main drivers of coral demise, but experimental evidence is clearly lacking [13,14]. Suggested mechanisms include contact-mediated effects such as allelopathy [15,16] and transmission of pathogens [17]. Water-mediated effects may also impact corals with the release of hydrophilic allelochemicals and dissolved organic carbon (DOC) feeding copiotroph and potentially virulent opportunistic microbes on the benthos and in the water column (i.e. the DDAM model: DOC, Disease, Algae and Microbes [13,18–21]). Although macroalgal assemblages shape the microbial and chemical composition of reef waterscapes [21], their effects on the coral microbiome can be inconsistent. For example, the microbiome of *Pocillopora damicornis* showed no variation in composition between algal- versus coral-dominated reefs in Fiji [22,23]. In another study, coral microbiome diversity and variability increased with increasing site-level algal cover in two islands of French Polynesia, even in the absence of direct contact [24]. Given the importance of microorganisms on holobiont health and the variable results produced by current studies, a better understanding of algal effects on the coral microbiome is needed to predict the resilience of coral communities as macroalgae proliferate.

Coral recruitment is a fundamental process for the replenishment of coral communities [11,25,26]. Macroalgal assemblages can create a major demographic bottleneck in coral recruitment [26,27]. In particular, an *in situ* study comparing recruitment success between algal- versus coral-dominated reefs suggests negative algal effects on larval settlement and post-settlement survival [12]. These effects have been related to alterations in the microbiome of microbenthic communities [26,28]. However, the links between macroalgal abundance, coral recruitment success and the microbiome of coral early life stages are poorly understood. In Fiji, the composition of the larval microbiome of *P. damicornis* between algal- versus coral-dominated reefs remained unchanged despite lower larval survival in algal-dominated seawater [22].

Furthermore, parental effects (i.e. effect of the parental phenotype on the phenotype or performance of the offspring) can influence the survival and growth of coral offspring. The extent of these parental effects varies depending on the reproductive strategy. For instance, in the spawner *Acropora tenuis* and the brooder *Porites astreoides*, parental effects can explain up to 17 and 94% of the variance in juvenile survival, respectively [29,30]. In brooding species, that internally produce larvae, maternal effects are expected to be stronger than in broadcast spawning species due to a greater maternal investment [31] and a vertical transmission of microbial symbionts [3,4]. The environmental stress experienced by the parents can lead to positive and negative cross-generational effects on offspring performance and has been related to altered maternal provisioning [32]. For example, the transfer of essential metabolites (e.g. lipids, amino acids) is reduced in eggs of heat-stressed parents in soft corals [33]. In addition, parents could differentially invest in their offspring by vertically transmitting distinct Symbiodiniaceae [30] and prokaryotic communities [5,34]. So far, negative parental effects in corals and other marine invertebrates, such as urchins, have essentially been demonstrated relative to climate-associated stressors [32–34]. Yet, given that macroalgae can alter both health and fecundity of corals, one might expect that negative parental effects relative to algal competition might jeopardize coral recruitment success. To our knowledge, only one study [22] has suggested algal-induced parental effects on corals. However, those effects were not associated with changes in the offspring microbiome. Thus, whether parental effects exist under coral–algal competition and whether they could influence offspring performance via effects on the larval microbiome represent major knowledge gaps in our understanding of the mechanisms by which macroalgae may prevent coral recovery.

In this work, we investigated the effect of dense macroalgal assemblages on the brooding coral *Pocillopora acuta*. We hypothesized that macroalgal assemblages may have negative parental and offspring environmental effects on coral recruitment and that these effects may be mediated by alterations in the coral and substrate microbiome. To test these assumptions, we conducted a manipulative field experiment in which we compared coral (i.e. adults and larvae) and microbenthic substrate microbiomes between algal-removed and algal-dominated bommies within a single fringing reef in Mo'orea, French Polynesia, to control for confounding reef-scale effects. Then, we assessed the relative influence of parental and offspring environmental effects on coral recruitment success by reciprocally exposing coral larvae from two parental origins (i.e. colonies originating from algal-removed versus algal-dominated bommies) to algal-removed and algal-dominated environmental conditions in a series of survival and settlement experiments.

## 2. Material and methods

### 2.1. Experimental field set-up

We conducted a manipulative field experiment between June and December 2020 in a shallow fringing reef lagoon (2–2.5 m deep) off the north coast of Mo’orea, French Polynesia (17°29*′*14.86*″* S, 149°53*′*0.76*″* W). In late June 2020, eight coral bommies (1–2 m in diameter) covered by dense macroalgal assemblages were randomly selected within an area of approx. 1000 m² (distance between adjacent bommies: approx. 10 m; electronic supplementary material, figure S1*a*,*b*). Bommies were randomly allocated to one of the two treatments: (i) macroalgae present (referred to as ‘algal-dominated’) and (ii) macroalgae removed (i.e. including canopy-forming holdfasts and understory species, referred to as ‘algal-removed’) (electronic supplementary material, figures S1*c*,*d* and 2*a*). Macroalgae were removed from their substratum manually with wire brushes taking care not to damage other benthic organisms or to alter the substrate topography. Their absence on algal-removed bommies was maintained on a monthly basis. Before each monthly maintenance, only algal juveniles or re-grown short-sized (<1 cm) algae were observed, indicating that this removal frequency was sufficient.

After algal removal, six colonies (approx. 12 cm in diameter) of the brooding coral *P. acuta* were transplanted on each experimental bommie and left untouched for 5–6 months, a period sufficient for parental effects to occur [32]. Colonies were collected across the fringing reef within a 200–300 m radius around the bommies. The experimental bommies were almost free of conspecifics. Only a single juvenile of *P. acuta* (*ca* 2 cm in size) was observed on one bommie in November 2020 (C.P.-S., personal observation). Colonies were chiselled at their base and secured on small (4 × 4 cm^2^) tagged plastic grids with two cable ties. Grids were then attached to the bommies with releasable cable ties and tagged mounting screws inserted into holes drilled into dead coral substratum using a pneumatic drill. This procedure was used, so colonies could be brought to the laboratory facilities for coral larval collection and re-attached to the bommies at their exact same position. Colonies were placed on substrate predominantly covered by bare substrate, crustose coralline algae (CCA) or thin (<5 mm) turf communities, without direct physical contact with macroalgae, although some periodic contact may have occurred due to water movement.

In August 2020, 11 aragonite tiles (3 × 3 × 1 cm^3^) were fixed on each bommie for the sampling of substrate microbiome and the coral recruitment experiments (electronic supplementary material, figure S2*a–c*). One hole was drilled at the centre of each tile for attachment to the dead coral substratum. Each tile was tagged and fixed on the bommies with a cable tie and a tagged mounting screw inserted into a hole drilled into dead coral substratum using a pneumatic drill. Similar to the coral colonies, tiles were placed without direct contact with macroalgae.

### 2.2. Benthic surveys

Benthic communities were quantified on each bommie by taking photographs of five randomly placed, 20 × 20 cm^2^ quadrats with an Olympus TG4 camera in June before algal removal and in both August and November 2020 after the monthly maintenance. Benthic categories included: bare substrate (i.e. the absence of macroorganisms but presumably colonized by microalgae), CCA, cyanobacteria, living hard coral, algal turf (i.e. mixed species assemblages of filamentous algae <1 cm in height) and macroalgae (upright and anatomically complex algae with canopy height >1 cm). Within the macroalgae category, eight genera or species were recorded: *Amansia rhodantha*, *Chnoospora* spp., *Dictyota bartayresiana*, *Halimeda* spp., *Lobophora* spp., *Padina boryana*, *Sargassum pacificum* and *Turbinaria ornata*. The percentge cover of each category was calculated for each quadrat from 25 random points using the software PhotoQuad [35]. For each category, the percentage cover corresponded to the number of points of that category divided by the total number of identifiable points in the quadrat.

### 2.3. Microbiome sampling

In November 2020, three fragments of three colonies from each bommie were sampled to characterize the microbiome of adult corals (electronic supplementary material, figure S2*b*). Coral fragments were broken off into 2 ml microtubes underwater and transported back at the laboratory into cool boxes with ice. Larvae brooded by these colonies were sampled on 19 and 20 November (fourth and fifth days after the new moon). Owing to logistic constraints, two colonies out of the three sampled for the adult microbiome were randomly selected on each bommie and brought to the laboratory. They were grouped by each treatment and isolated into individual plastic containers (approx. 25 l) placed inside a large water tank filled with 220 l of seawater collected approx. 50 cm above the bommies from their respective treatment. Seawater was recirculated in each closed system using one pump (approx. 2000 l h^−1^; EHEIM compact + 3000 pump), and water temperature was maintained at approx. 27°C using heaters. Container outflows were covered by a 200 μm mesh net around 23.00 to prevent larval escape. Larvae were released throughout the night and, early morning (between 05.00 and 07.00), 30 larvae of each colony were collected with sterile pipette tips. One colony of each treatment did not release larvae. Each fragment and larvae of each colony were rinsed three times with 0.22 µm filtered sterilized seawater (FSW), transferred into 2 ml cryotubes with 1 ml of DNA/RNA Shield (Zymo Research). In addition, one water sample (5 l) from each water tank was filtered onto a 0.22 µm filter. Filters were cut with sterile tools, and pieces were transferred into 2 ml cryotubes with 1 ml of DNA/RNA Shield. In December 2020 (2 days before the settlement experiment), three tiles per bommie were randomly sampled to characterize the substrate microbiome. Tiles were placed into individual sterile Whirl-Pak bags underwater and transported in a cool box with ice to the laboratory. Cryptic and exposed surfaces of each tile were sampled independently. After rinsing with FSW, surfaces were sampled first by scraping with a sterile scalpel and second by swabbing with a sterile cotton swab. Scrapings and cotton swab heads of each surface were placed into 2 ml cryotubes with 1 ml of DNA/RNA Shield. DNA/RNA Shield-containing cryotubes were stored at −20°C until DNA extraction.

### 2.4. Sequencing of the 16S rRNA gene and metabarcoding data processing

DNA was extracted using a ZymoBIOMICS DNA Miniprep kit (Zymo Research, USA, D4300). Both sampled materials and 1 ml of DNA/RNA Shield were placed in bead tubes. Extractions were then performed from the lysate according to manufacturer instructions for samples stored and lysed into DNA/RNA Shield. After extraction, samples were stored at −20°C and dried with a centrifugal evaporator (EZ-2 series, Genevac) for safe shipment to Genome Quebec (Montréal, Canada) for 16S rRNA marker gene amplification by PCR and sequencing. Unfortunately, four coral samples were damaged during shipment. The hypervariable region V3–V4 of the 16S rRNA gene was amplified using the universal primers 341F (5′-CCTACGGGNGGCWGCAG-3′) and 805R (3′-GACTACHVGGGTATCTAATCC-5′), suggested for marine bacteria and some archaea [36], and amplicons were sequenced on an Illumina NovaSeq 6000 sequencer. Paired-end (2 × 250 bp) reads were processed using DADA2 [37] on R (v. 4.2.3) to generate amplicon sequence variants (ASVs). First, reads with ambiguous N bases were discarded, and the primers were removed using the Cutadapt program [38]. Reads were filtered out if their length was not comprised between 200 and 250 bp and if they contained bases with a quality score inferior to 2 or more than 3 expected errors. Sequences were denoised based on a modified error rate estimation function, by altering loess arguments (weights and span) and enforcing monotonicity, more suitable for NovaSeq data. After reads merging and ASV inference, chimeric sequences and those detected in a single sample were removed. Taxonomy was assigned from phylum to genus levels using the Silva reference database (v. 138.1). The sequence table, taxa table and metadata were then imported in the *phyloseq* R package [39]. Finally, low abundance ASVs (i.e. at least 20 reads in 5% of the samples), eukaryotic ASVs (chloroplast and mitochondria) and contaminants (*decontam* R package with prevalence method using our *n* = 9 blank samples [40]) were removed from the dataset prior to analysis.

### 2.5. Coral recruitment experiments

To determine the relative influence of parental and offspring environmental effects on coral recruitment processes, offspring produced by each parental origin was reciprocally exposed to algal-removed and algal-dominated environmental conditions in a series of experiments (electronic supplementary material, figure S2*c*). In the larval survival experiment, newly released larvae were collected on 18 December 2020 (fifth day after the new moon) as described earlier, pooled by their parental origin and deployed in a full factorial design into 1 l glass jars filled with 400 ml of seawater freshly collected approx. 50 cm above algal-removed or algal-dominated bommies. Each jar held 10 larvae, and there were 12 replicated jars per combination of factors: (i) algal-removed parental origin × algal-removed seawater, (ii) algal-removed parental origin × algal-dominated seawater, (iii) algal-dominated parental origin × algal-removed seawater, and (iv) algal-dominated parental origin × algal-dominated seawater. Jars were randomly interspersed in a heat-regulated water bath. Light was provided by two lamps (ViparSpectra aqualight system 165W) set approx. 45 cm above the jars on a 12 : 12 h photoperiod with an irradiance of approx. 60 µmol m^−2^. Seawater was changed every 2 days (30% of 400 ml, that is, 120 ml). Temperature was recorded every 15 min with two HOBO Pendant^®^ loggers inside the water baths. In addition, temperature, pH, conductivity and O_2_ saturation were monitored twice daily in eight randomly selected jars with probes. At day 6, the number of surviving larvae was counted in each jar using a binocular magnifier. Larvae were considered alive if swimming after gentle pipette aspirations.

In the larval settlement and post-settlement survival experiments, newly released larvae were collected on 20 December 2020 as described earlier, pooled by their parental origin and deployed in a full factorial design into 1 l glass jars, each containing 800 ml of seawater and one tile originating from either algal-removed or algal-dominated bommies. A dowel was inserted through the central hole of each tile, so their cryptic (underside) surface was accessible to larvae. Each jar held 10 larvae, and there were 12 replicated jars per combination of factors: (i) algal-removed parental origin × algal-removed seawater and tile (i.e. hereafter ‘offspring environment’), (ii) algal-removed parental origin × algal-dominated offspring environment, (iii) algal-dominated parental origin × algal-removed offspring environment, and (iv) algal-dominated parental origin × algal-dominated offspring environment. Jars were maintained in the water bath as described earlier under the same environmental conditions, and water physiochemical parameters were monitored in the same manner. After 24 h, the number of settled larvae were counted on the exposed (upperside), cryptic and vertical surfaces of the tiles and individually mapped. After the initial mapping, tiles with settled larvae were returned to their tagged field positions and re-examined at days 7 and 21 to estimate early post-settlement survival. Larval survival and settlement were expressed as percentages of the initial number of larvae added to each jar. Post-settlement survival was expressed as percentage of the number of coral settlers counted after 24 h on each tile.

### 2.6. Statistical analysis

#### 2.6.1. Benthos data

Variations in benthic assemblages before and after algal removal were assessed by permutational multivariate analysis of variance (PERMANOVA, adonis2:*vegan* and 999 permutations [41]). Specifically, they were assessed: (i) between algal treatment (fixed factor with two levels: algal-dominated versus algal-removed bommies) before removal of macroalgae and (ii) between algal treatment and time (fixed factor with two levels: August versus November) and their interaction after the removal of macroalgae. Benthic community composition between algal treatment was visualized using principal coordinate analysis (PCoA) on Bray–Curtis dissimilarities of untransformed percentage cover data. We used the function envfit:*vegan* [41] to overlay main benthic categories on the PCoA plot and identify the categories which contributed significantly to differences in benthic community composition (999 permutations).

#### 2.6.2. Microbiome data

Coral and substrate microbiomes were analysed separately. Alpha diversity (Shannon diversity index) of coral and substrate microbiomes was measured on rarefied count data using the rarefy_even_depth:*phyloseq* function [39]. Differences in alpha diversity were tested using a linear mixed-effects model (LME; *lme4* [42] and *emmeans* R packages [43]). For coral microbiome, life stage and algal treatment and their interaction were included as fixed factors and colony nested within bommie as random effects. For substrate microbiome, surface and algal treatment and their interaction were used as fixed factors and bommie as random effect. Beta diversity statistics (i.e. microbiome composition and variability) were conducted on centred log-ratio (CLR)-transformed count data (package *microbiome* [44]) and Euclidian distances. Compositional differences in coral and substrate microbiomes were tested by PERMANOVA (999 permutations). Microbiome variability (beta dispersion) was investigated with the betadisper:*vegan* function and tested with a permutation test (999 permutations). Both tests were run with the same fixed factors as for the alpha diversity analyses, excluding the interaction terms for beta dispersions. Coral and substrate microbiome compositions were visualized by principal component analysis (PCA).

To identify which microbial taxa contributed the most to microbiome compositional differences, we used a supervised classification algorithm random forest (package *caret* [45]). Models were trained with 500 trees following a K-folds cross-validation scheme (sixfold, trainControl:*MLmetrics* package [46]). Model performance was evaluated by its accuracy score, and the best predictors were retrieved by their increase in mean squared error (IncMSE) score (varImp:*caret* [45]). First, we conducted the analysis at the family level across combined levels of life stage/algal treatment for the coral microbiome and of surface/algal treatment for the substrate microbiome. We selected the 20 most explaining families to build two-way heatmaps from their mean CLR-transformed abundances using Euclidian distances and Ward’s minimum variance method. Then, to specifically identify the ASVs best explaining differences between algal treatments, we ran random forests on the microbiomes of coral adults, coral larvae and cryptic and exposed surfaces independently. Analysis of compositions of microbiomes with bias correction (ANCOM-BC) package [47] was used for differential abundance analysis on the microbial families and the 100 top-ranked ASVs. Differential abundances were considered significant if adjusted *p*-values were <0.05. For cryptic and exposed surfaces, significant ASVs were shown if they had a log-fold change >2 or <−2 due to the high number of significantly different ASVs. A positive log-fold change indicates an increase in ASV abundance in samples from algal-dominated bommies relative to algal-removed bommies, whereas a negative log-fold change indicates a decrease.

#### 2.6.3. Coral recruitment experiment data

Survival and settlement were calculated as the number of living larvae (swimming and settled) and the number of larvae that had settled on tiles, respectively, divided by the initial number of larvae added to the jar. For post-settlement survival, the number of living recruits was divided by the initial number of settlers. Generalized linear mixed-effects models (GLME; glmer:*lme4* package [42]) with a binomial distribution were used to test differences in the probability of larval survival, settlement and post-settlement. Survival data were modelled with parental origin (i.e. larvae brooded by parents originating from algal-dominated versus algal-removed bommies) and seawater (i.e. algal-dominated versus algal-removed bommies) and their interaction as fixed factors. Settlement data were, first, analysed by pooling all tile surfaces (i.e. exposed, cryptic and vertical) and using parental origin and offspring environment (i.e. seawater and tile from algal-dominated versus algal-removed bommies) and their interactions as fixed factors and bommie as a random factor. To assess surface-specific differences, a second model was run adding surface as a fixed factor and tile nested within the bommie as a random factor. Owing to low settlement on exposed and vertical tile surfaces, post-settlement survival was modelled using cryptic surface data by adding time as a fixed factor and bommie as a random factor. Post-settlement survival data were analysed with parental origin, offspring environment (i.e. seawater, tile and nursery area from algal-dominated versus algal-removed bommies), time (i.e. day 7 versus 21) and their interactions as fixed factors and bommie as a random factor. Pairwise comparisons were conducted when fixed effects were significant using Tukey’s test on marginal means (*emmeans* package [43]). Model assumptions of homogeneity of variance, normal distribution of the residuals and the absence of overdispersion were checked with the *DHARma* package [48].

## 3. Results

### 3.1. Benthic community composition

Benthic community composition did not differ between algal treatments before the start of the experiment (PERMANOVA, algal treatment: *R*^2^ = 0.02, *p* > 0.05). After algal removal, community composition varied significantly between algal treatments and remained distinct throughout the experiment (PERMANOVA, algal treatment: *R*^2^ = 0.59, *p* = 0.001; time: *R*^2^ = 9 × 10^−4^, *p* > 0.05; treatment × time: *R*^2^ = 0.05, *p* = 0.16; electronic supplementary material, figure S3*a*). Macroalgae, turf, bare substrate, CCA and hard corals significantly contributed to differences in benthic community (*p* < 0.05). Specifically, algal-removed bommies were dominated by bare substrate (approx. 44% cover) and turf (approx. 28% cover), whereas algal-dominated bommies were dominated by macroalgae (approx. 68% cover), with the brown macroalgae *T. ornata* (approx. 39% cover) and *D. bartayresiana* (approx. 11% cover) as the two most abundant species (electronic supplementary material, figure S3*b*,*c*).

### 3.2. Coral microbiome composition

**Figure 1.**
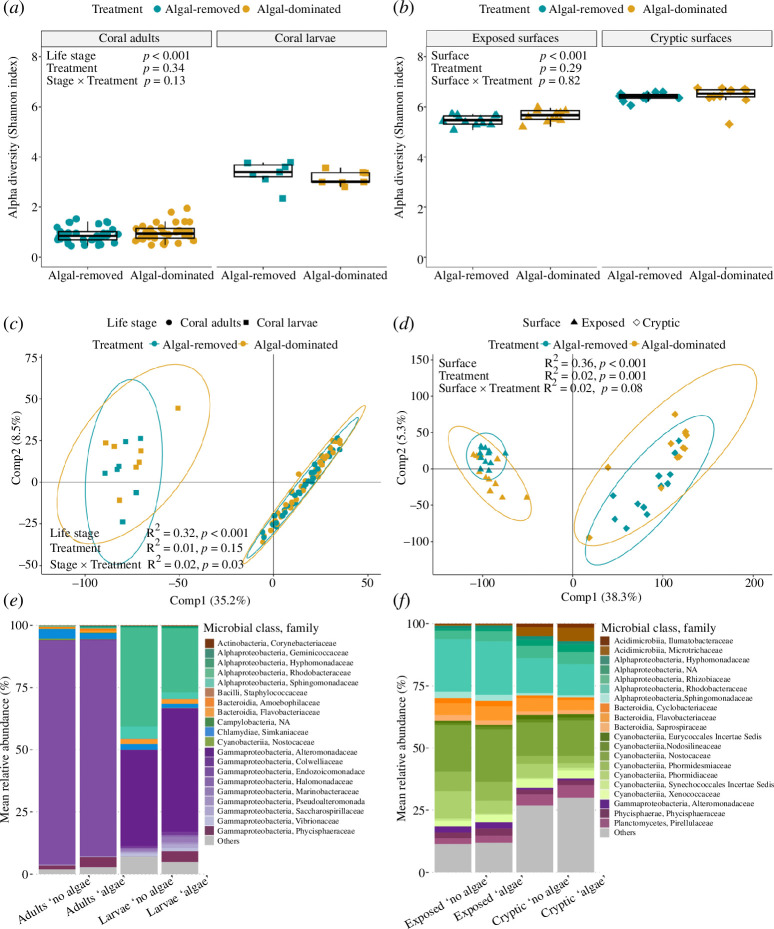
Microbiome diversity and composition. Alpha diversity of (*a*) coral adults and larvae and of (*b*) cryptic and exposed surfaces from algal-removed and algal-dominated bommies. Effects of coral life stage/surface and algal treatment on diversity were tested using linear mixed-effects models with details in electronic supplementary material, table S2. Beta diversity of (*c*) coral adults and larvae and (*d*) cryptic and exposed surfaces from algal-removed and algal-dominated bommies. Variations of microbiome composition were tested by PERMANOVA (999 permutations). PCA was run on centred log-ratio (CLR)-transformed amplicon sequence variant (ASV) abundances with Euclidian distances. Ellipses represent the spread of data points at a 95% confidence level. Top 20 most abundant microbial families across (*e*) coral adults and larvae and (*f*) cryptic and exposed surfaces from algal-removed and algal-dominated bommies.

The final 16S rRNA gene dataset data resulted in 4474 ASVs and 26 514 802 reads across 132 samples (electronic supplementary material, figure S4 and table S1). Alpha diversity of coral microbiomes significantly differed between adults and larvae (LME, coral life stage: *p* < 0.001), but not between algal treatments (*p* > 0.05; figure 1*a*; electronic supplementary material, table S2*a*). Larval microbiomes (emmean = 3.43 ± 0.09) were nearly three times more diverse than adult microbiomes (emmean = 1.02 ± 0.05). Coral prokaryotic community significantly varied across coral life stages (PERMANOVA, coral life stage: *p* < 0.001; figure 1*c*), but the effect of coral life stage differed between algal treatments (coral life stage × algal treatment: *p* < 0.05). Specifically, the algal treatment significantly structured the larval microbiome (pairwise PERMANOVA, *p* = 0.01), but not the adult microbiome (*p* > 0.05; electronic supplementary material, table S3*a*). Microbiome variability was higher in larval compared to adult stages regardless of the algal treatment (PERMDISP, coral life stage: *p* < 0.001; algal treatment: *p* > 0.05; electronic supplementary material, figure S5*a* and table S4*a*).

The coral adult microbiome was largely dominated by the Endozoicomonadaceae family (class Gammaproteobacteria), accounting for 88.9% of the microbial sequences (figure 1*e*; electronic supplementary material, figure S6*a*). Comparatively, the coral larval microbiome harboured a greater diversity of Gammaproteobacterial families, such as Alteromonadaceae (e.g. *Alteromonas*, *Salinimonas*), accounting for nearly half of the bacterial community (45.6%), followed by Marinobacteraceae (1.4%, *Marinobacter*) and Vibrionaceae (1.1%, *Vibrio*; electronic supplementary material, figure S6*a*). Numerous Alphaproteobacterial families, such as Rhodobacteraceae (33.6%, e.g. *Maribius*, *Limmimaricola*) and Sphingomonadaceae (3.8%, *Erythrobacter*), were also highly abundant in coral larvae. Overall, the respective dominant bacterial families in the adult and larval microbiome displayed little differential abundance between algal treatments (electronic supplementary material, figures S6*a* and S7*a*). A single family, Flavobacteriaceae (class Bacteroidia), was significantly higher in the adult microbiome from algal-dominated bommies (ANCOM-BC, *p*-adj < 0.05).

### 3.3. Substrate microbiome composition

Substrate microbiome diversity did not differ between algal treatments (LME, *p* > 0.05), but significantly differed between surfaces (*p* < 0.001; figure 1*b*; electronic supplementary material, table S2*b*). The microbiome of the cryptic surface (emmean = 6.78 ± 0.06) was more diverse than that of the exposed surface (emmean = 5.80 ± 0.06). The composition of the substrate microbiome significantly differed between surface and algal treatment (PERMANOVA, surface: *p* < 0.001; algal treatment: *p* < 0.001; surface × algal treatment: *p* > 0.05; figure 1*d*; electronic supplementary material, table S3*b*). The substrate microbiome variability did not vary between algal treatments, but significantly differed between surface (PERMDISP, surface: *p* < 0.001; algal treatment: *p* > 0.05; electronic supplementary material, figure S5*b* and table S4*b*). Specifically, the microbiome variability of cryptic surfaces was significantly higher than that of exposed surfaces.

Microbiomes of exposed and cryptic surfaces were dominated by the classes Cyanobacteria, Alphaproteobacteria and Bacteroidia (figure 1*f*). Nostocaceae was the most abundant Cyanobacterial family representing 21.1 and 15.7% of the microbial sequences on exposed and cryptic surfaces, respectively, followed by Phormidiaceae, which was also more abundant on exposed surfaces. Rhodobacteraceae (e.g. *Ruegeria*, *Limibaculum*; electronic supplementary material, figure S6*b*) and Rhizobiaceae were dominant families of the Alphaproteobacteria class. The former accounted for 22.1 and 14.3% of the microbial sequences on exposed and cryptic surfaces, respectively. Three families of Bacteroidia, in particular, the Flavobacteriaceae (e.g. *Muricauda*), were abundant. In addition, both exposed and cryptic surfaces contained ASVs belonging to the Planctomycetes, Phycisphaerae and Acidimicrobiia classes. No microbial family was significantly differentially abundant between algal treatments on exposed surfaces (ANCOM-BC, *p*-adj > 0.05). On cryptic surfaces, the relative abundance of 14 families significantly varied with algal treatment, including Stappiaceae, Phormidiaceae, Cellvibrionaceae and Tenderiaceae, which were lower on algal-dominated bommies relative to algal-removed bommies (ANCOM-BC, *p*-adj < 0.05; electronic supplementary material, figure S7*b*).

### 3.4. Differential abundance analysis of amplicon sequence variants between algal treatments

**Figure 2.**
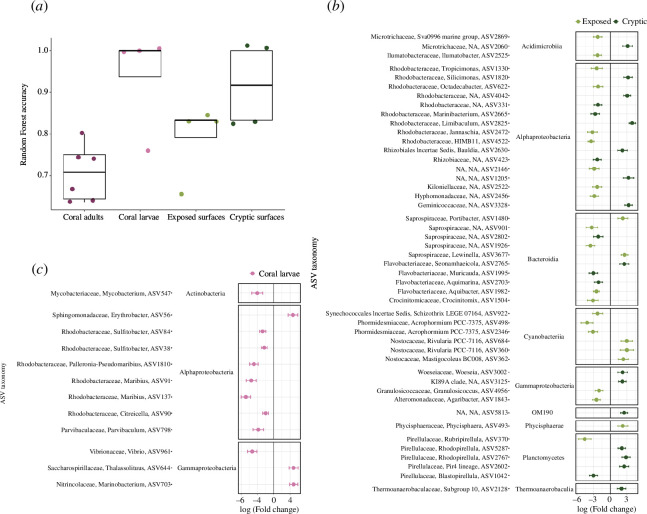
Microbial amplicon sequence variants (ASVs) associated with algal-removal treatment. (*a*) Random forest accuracy for classifying samples relative to the algal-removal treatment. Significantly differentially abundant ASVs (ANCOM-BC, *p-*adj < 0.05) between algal-dominated versus algal-removed bommies within (*c*) coral larval samples and (*b*) exposed and cryptic surfaces. No significantly differentially abundant ASVs were detected in coral adult samples. Fold changes were calculated relative to the mean changes in the algal-removed treatment. For the surface microbiomes, only ASVs with a log-fold change >2 or <−2 are included. ASVs are grouped by family, genus and class. A positive log-fold change indicates an increase in ASV abundance in samples from algal-dominated bommies relative to algal-removed bommies, whereas a negative log-fold change indicates a decrease.

Random forests achieved a strong (>70%) classification accuracy for all sample types (figure 2). However, no significantly differentially abundant ASV between algal treatments was detected in the coral adult microbiome. In the coral larval microbiome, 12 ASVs differed significantly between algal treatments (ANCOM-BC, *p*-adj < 0.05; figure 2*c*). Most were affiliated to the Alphaproteobacteria. Six belonged to the Rhodobacteraceae, including two *Maribius*, one *Palleronia–Pseudomaribius* and two *Sulfitobacter*, and significantly decreased in larvae brooded by corals from algal-dominated bommies relative to algal-removed bommies. Interestingly, four of these taxa were undetected in the coral adult microbiome but present in the coral larval collection tank (electronic supplementary material, table S5). Other decreasing ASVs belonged to the genera *Mycobacterium* and *Vibrio*, the latter being closely related to *Vibrio parahaemolyticus* (ASV961; electronic supplementary material, table S6). In contrast, ASVs enriched in larvae brooded by corals from algal-dominated bommies were affiliated with the genera *Erythrobacter*, *Thalassolituus* and *Marinobacterium*. In addition, an ASV which was identified as *Vibrio coralliilyticus* (ASV2412; electronic supplementary material, table S6), a well-described coral pathogen [1], was detected in coral larvae microbiomes, but its abundance did not differ between algal treatments.

Comparatively, a high number of ASVs in the substrate microbiomes differed significantly between algal treatments (i.e. 37 and 30 ASVs for exposed and cryptic surfaces, respectively). Therefore, we only showed the significant ASVs with a log-fold change >2 or <−2 (figure 2*b*). On the exposed surfaces, a majority of ASVs were depleted on algal-dominated bommies relative to algal-removed bommies. These were affiliated to the Acidimicrobiia (e.g. *Ilumatobacter*), Alphaproteobacteria (e.g. *Tropicimonas*, *Jannaschia*), Gammaproteobacteria (*Granulosicoccus*, *Agaribacter*) and Planctomycetes (*Rubripirellula*). Among the Bacteroidia, ASVs affiliated to the genera *Portibacter* and *Lewinella* (Saprospiraceae) were significantly enriched on the exposed surface of algal-dominated bommies relative to algal-removed bommies, while *Aquibacter* (Flavobacteriaceae) and *Crocinitomix* (Crocinitomicaceae) displayed the opposite trend. ASVs belonging to the Cyanobacteriia were only flagged in the microbiome of the exposed surface. For example, the ASV *Acrophormium PCC-7375* (Phormidesmiaceae) was significantly depleted, while *Rivularia PCC-7117* (Nostocaceae) was significantly enriched on algal-dominated bommies relative to algal-removed bommies. On the cryptic surface, several ASVs associated with the Alphaproteobacteria and Planctomycetes were significantly enriched in algal-dominated bommies, such as those belonging to the genera *Silicimonas*, *Limibaculum* (Rhodobacteraceae), *Bauldia* (Rhizobiales Incertae Sedis), *Rhodopirellula* and *Pir4 lineage* (Pirellulaceae). In contrast, seven ASVs were depleted on the cryptic surface of algal-dominated bommies. These ASVs belonged to the genera *Marinibacterium* (Rhodobacteraceae), *Muricauda*, *Aquimarina* (Flavobacteriaceae) and *Blastopirellula* (Pirellulaceae).

### 3.5. Coral recruitment experiments

**Figure 3.**
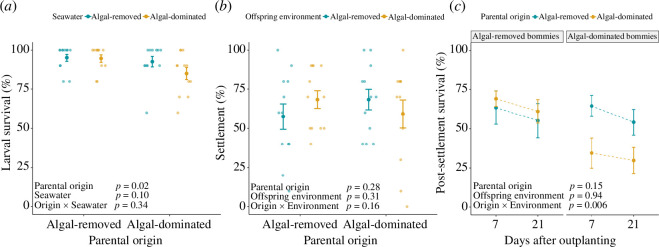
Coral recruitment experiments. (*a*) Survival (mean ± s.e., *n* = 12) of larvae brooded by corals from algal-removed and algal-dominated bommies after 6 days in seawater from each algal treatment. (*b*) Settlement (mean ± s.e., *n* = 12) of larvae brooded by corals from algal-removed and algal-dominated bommies on tiles from algal-removed and algal-dominated bommies after 24 h. (*c*) Post-settlement survival (mean ± s.e., *n* = 12) of coral settlers produced by corals from algal-removed and algal-dominated bommies 7 and 21 days after out-planting on algal-removed and algal-dominated bommies. Statistical significance was assessed with generalized linear mixed-effects models (GLME) using binomial distribution; detailed results can be found in electronic supplementary material, table S8. Summary table of the data can be found in electronic supplementary material, table S9.

Water physiochemical conditions of larval survival and settlement experiments were similar across algal treatments (electronic supplementary material, table S7; Kruskal–Wallis, *p* > 0.05). Parameters averaged 27.92°C (±0.63) for temperature, 8.24 (±0.09) for pH, 29.11 psu (±1.03) for salinity and 87.34% (±5.09) for O_2_ saturation. The parental origin significantly influenced larval survival (GLME, *p* = 0.02; figure 3*a*; electronic supplementary material, table S8). Specifically, the percentage of larval survival on all bommies declined from 94.8% (±1.7) for larvae brooded by parents from algal-removed bommies to 88.8% (±2.6) for larvae brooded by parents from algal-dominated bommies, representing a reduction of 6.3% (electronic supplementary material, table S9). The analysis revealed no significant seawater effect or interaction between parental origin and seawater on larval survival.

When considering settlement on all surfaces, there were no parental or offspring environmental (i.e. seawater and tile) effects or interaction between these factors on larval settlement, which averaged 63.3% (±3.7) (figure 3*b*; electronic supplementary material, table S8*b*). When adding tile surface to the model, there was a significant three-way interaction among parental origin × environment × tile surface (GLME, *p* < 0.001). *Post hoc* tests revealed that larvae brooded by corals on algal-dominated bommies settled less on cryptic surfaces from algal-dominated bommies compared to those from algal-removed bommies (electronic supplementary material, table S8*c* and figure S8). In contrast, the opposite was found for vertical tile surfaces. Therefore, larvae brooded by corals from algal-dominated bommies preferred to settle on slightly more exposed surfaces of algal-dominated bommies.

There was a significant interaction between parental origin and environmental factors on post-settlement survival (GLME, *p* = 0.006; figure 3*c*; electronic supplementary material, table S8*c*). *Post hoc* tests revealed a significant environmental effect on post-settlement survival for larvae brooded by parents from algal-dominated bommies only. The percent survival of recruits produced by parents from algal-dominated bommies declined from 69.1% (±4.9) on algal-removed bommies to 34.5% (±9.6) on algal-dominated bommies at day 7 and from 61.1 (±7.5) to 29.8% (±8.3) at day 21, representing reductions of 50.1 and 51.2% on days 7 and 21, respectively. In contrast, post-settlement survival of larvae brooded by parents from algal-removed bommies did not vary between algal treatments and averaged 61.5 (±6.2) and 52.7% (±6.8) for days 7 and 21, respectively (electronic supplementary material, table S9).

## 4. Discussion

By impairing coral holobiont health and recruitment success, macroalgae constitute an extensive threat to coral replenishment processes. Here, we demonstrate that dense macroalgal assemblages alter the coral larval microbiome and have negative parental and offspring environmental effects on coral recruitment. Our study is the first to link parental effects under coral–algal competition with alterations in the coral larval microbiome and to reveal interacting parental and offspring environmental effects on coral early life stages. It provides important new knowledge on the response of coral microbiomes to macroalgal dominance and how it relates to offspring performance.

Our work showed that dense macroalgal assemblages did not significantly alter the microbiome of adult corals at the scale of coral bommies within a single fringing reef. Despite the large differences in macroalgal cover between the algal-removed and algal-dominated bommies (i.e. 4% versus 68%) and the five-month exposure to each treatment, the diversity, variability and composition of the coral adult microbiome did not significantly vary between the algal treatments. These results disagree with several studies suggesting that macroalgae can alter the coral microbiome [10,15,24]. However, they may be explained by the fact that our sampled colonies were initially placed without direct physical contact with macroalgae. Many of the mechanisms by which algae affect corals, including the release of allelochemicals [15,16] and dissolved organic matter [18,19], operate on small spatial scales. For example, the transmission of coral pathogens is likely to require direct contact [49]. These results could also be related to the identity of the coral host itself. Pocilloporids have a remarkable capacity to maintain their microbiome under macroalgal competition [22] and abiotic disturbances [50,51]. Ziegler *et al.* [50] proposed that microbiome flexibility is host specific with Pocilloporids being ‘microbiome regulators’, capable of some microbial regulations while keeping a constant microbiome [50]. This property may be related to their opportunist life-history strategies characterized by high recruitment rates and fast growth [52]. Although a lack of microbiome flexibility may hinder the acclimatization potential of corals [53], a stable microbiome could enhance coral resilience to rapid environmental changes and microbial pathogens [23,51]. For example, microbiome stability was associated with unaltered anti-pathogen activity against *V. coralliilyticus* in adult *P. damicornis* corals from both coral-dominated marine protected areas and macroalgal-dominated fished reefs in Fiji [23]. In our study, the relative abundance of Vibrionaceae in adult *P. acuta* corals was not significantly influenced by the algal treatment. In contrast, corals from both algal-removed and algal-dominated bommies were largely dominated (>85% relative abundance) by bacteria from the Endozoicomonadaceae family. This family of bacteria likely plays critical roles in the health and resilience of coral holobionts of different coral species, including Pocilloporids [23,54].

The coral larval microbiome was compositionally distinct and more diverse and variable compared to the adult microbiome. These ontogenetic changes are associated with a ‘winnowing process’ [7] and match with the previous studies on corals [4–6]. The coral larval microbiome was dominated by the Rhodobacteraceae and Alteromonadaceae families. These families have been detected in high abundance in larvae of *P. acuta* [4] and other coral species [3,6]. Importantly, our study shows that the composition of the coral larval microbiome differed significantly between larvae brooded by corals from algal-removed and algal-dominated bommies. Our knowledge of the processes by which bacterial communities are acquired during the larval stage is still very limited [2–4]. However, in the closely related brooding coral *P. damicornis*, the establishment of bacterial communities in the offspring is driven by both parental and planulation environments, with the majority of uptake occurring horizontally (i.e. from the environment [5]). Similarly, in *P. acuta*, larvae shared a minority of their ASVs with their parents [4]. Since our colonies were maintained in seawater from their respective treatment during larval release, the relative importance of parental versus planulation environments on larval microbes cannot be separated. Larvae were collected less than 8 h upon emergence and rinsed in FSW three times before preservation. In a similar time frame, vertical transmission of bacteria can occur in the form of bacterial aggregates in newly released *P. acuta* larvae [4]. Likewise, bacterial cells in the coral *P. astreoides* were detected in the ectoderm of larvae in less than an hour after release constituting the vertically transmitted microbiome [3]. Therefore, it is likely that, in our study, both parental and planulation environments drove the differences in coral larval microbiome between algal treatments.

ASVs enriched in larvae brooded by parents from algal-dominated bommies were affiliated to the genera *Erythrobacter*, *Thalassolituus* and *Marinobacterium*. These bacteria have previously been associated with coral disease and coral–algal competition [55,56]. In contrast, the microbiome of these larvae was depleted in putative beneficial bacteria including members of the Rhodobacteraceae and *Vibrio* spp. For example, *Sulfitobacter* and *Vibrio* can produce and/or degrade dimethylsulfoniopropionate (DMSP) [57]. DMSP and its breakdown products (e.g. DMS) play major roles in coral health as antioxidants and antibiotics. These bacteria are also crucial for carbon and nitrogen acquisition, including *V. parahaemolyticus* [6,58,59]. While some *Vibrio* species like *V. coralliilyticus* and *V. parahaemolyticus* might also constitute opportunistic pathogens [1,59], *Vibrio* spp. can form mutualistic relationship with corals by providing nutrients and preventing bacterial colonization [60,61]. The loss of these beneficial bacteria could compromise larval physiology and defence through a decrease in their nutritional and protective functions. Remarkably, the relative abundance of the Alteromonadaceae family was not impacted by the algal treatment, demonstrating a strong host–microbe association. Some *Alteromonas* strains provide nitrogen sources [6,58], participate in larval settlement induction [25] and prevent pathogenetic invasion [62].

Macroalgal assemblages significantly structured the substrate microbiome, as previously demonstrated [26]. Microbial communities on algal-dominated bommies likely responded to macroalgal-induced changes in water chemistry. For example, algal-derived dissolved organic matter differs in composition from that of corals influencing the microbial structure of biofilm and bacterioplankton communities [20,63]. These labile and energy-rich algal exudates typically favour the growth of copiotrophic bacteria potentially detrimental to corals [20,21]. On exposed surfaces, certain bacteria enriched on algal-dominated bommies have been associated with coral diseases, including the genera *Lewinella* and *Rivularia* and the family Saprospiraceae [24,56]. Similarly, cryptic surface microbiomes from algal-dominated bommies harboured bacterial opportunists of corals, such as the genus *Limibaculum* [64], and several bacterial classes enriched on algal-dominated bommies have been negatively correlated with coral settlement, including Thermonanaerobaculia and Planctomycetes [28]. On algal-dominated bommies, several bacterial families and genera decreased relative to algal-removed bommies, potentially due to changes in competitive dynamics between microorganisms within microbenthic microbiomes [65].

After 6 days of exposure to seawater from algal-removed or algal-dominated bommies, mean larval survival remained high (>80%) in both algal treatments. Such high survival rates are consistent with the previous studies showing that Pocilloporid larvae can survive in the plankton for over 100 days [66]. Nevertheless, we found a significant effect of parental origin on larval survival. Larvae produced by parents from algal-dominated bommies had their survival reduced by approx. 6% relative to those produced by parents from algal-removed bommies. Similar and even stronger cross-generational effects of algal dominance on larval survival have been demonstrated when comparing larval survival from a coral-dominated marine protected area and a macroalgal-dominated fished area in Fiji [22]. Since the microbiome composition of larvae significantly differed between parental origins, these effects could be due to a compromised larval microbiome. As discussed earlier, larvae brooded by parents from algal-dominated bommies harboured a microbiome enriched with opportunistic bacteria and depleted in beneficial bacteria, which could have increased their mortality in the pre-settlement stage. At 6 days old, which corresponds to the duration of larval exposure to the algal treatments, coral larvae also extensively rely on their parental reserves [67]. Since reproduction is a costly process [9], algal-stressed parents may have faced a physiological trade-off in which they re-allocated energy for their own metabolism and defence rather than towards larval provisioning, thereby influencing their offspring survival. For example, bleached corals alter the quality and/or quantity of metabolites transferred to their larvae [33]. We failed to detect any effect of the environment (in this case, water origin) on larval survival. Previous laboratory-based studies found that polar algal compounds [15] and/or algal-associated microbes [18] could significantly decrease larval survival, contradicting our results. By using seawater, where algal dissolved compounds and microbes occur under natural concentrations, our result provides limited evidence of water-mediated effects on larval survival (but see [22]). However, these effects may have been underestimated as seawater was collected in the upper water column (approx. 50 cm) above the bommies and not near algal surfaces.

While macroalgae often prevent coral larval settlement in field and laboratory experiments [26,27], we found no parental and offspring environmental effects on coral larval settlement. Larvae settled equally on tiles from both algal treatments, although, by avoiding settling on tiles from algal-dominated bommies, they could have escaped the enhanced post-settlement mortality. This result occurred in spite of significant differences in substrate microbiome between algal treatments, suggesting that the substrate microbiome was not indicative of the negative environment for the larvae. It is possible that this result is due to the presence of positive cues (e.g. CCA) and/or limited abundance of negative cues (e.g. macroalgae) on the settlement tiles. After four months of conditioning, allowing for a mixed benthic community of settlement inducers and inhibitors [68], cryptic surfaces of settlement tiles were covered by CCA, a well-known coral settlement cue [69] and not densely covered by macroalgae (electronic supplementary material, figure S9). In addition, Pocilloporids are rather unselective of their microhabitats during settlement [70]. In contrast, algal-induced parental effects interacted with offspring environmental effects to affect the survival of coral recruits. Post-settlement survival was only reduced on algal-dominated bommies for recruits produced by parents from algal-dominated bommies. Such interaction may occur if algal-induced environmental effects coincide with the vulnerable state of recruits owing to their (i.e. algal-dominated) parental origin and/or if negative algal-induced parental effects are offset by the positive effects of the algal-removed environment. Our results do not allow us to distinguish between these two scenarios. However, the altered microbiome of coral larvae produced by parents from algal-dominated bommies is consistent with an increase in their vulnerability to detrimental (e.g. algal-dominated) environments.

By further supporting the negative effects of macroalgae on corals, our study adds further incentives to reduce macroalgal abundance on coral reefs. Because the conventional management actions of herbivore protection and water quality management are often insufficient to prevent coral–macroalgal phase shifts, physical removal of macroalgae has been suggested as a valuable intervention to rehabilitate degraded reefs [71]. Manually removing macroalgae (i.e. ‘sea-weeding’) can promote local-scale coral recruitment and recovery [72,73]. Our findings suggest that, if algae are removed from the vicinity of sexually mature adults for a few months prior to larval release, these adults could produce a more robust offspring and enhance coral recruitment, even on algal-dominated reefs. Future research should determine whether the positive parental effects of macroalgal removal on coral recruitment persist in the long term and whether similar effects occur in other coral species, in particular broadcast spawners.

## 5. Conclusion

As macroalgae proliferate and threaten reef health [8,12,14], it is essential to characterize their effects on the microbial dynamics of coral development stages and their habitats to predict future coral community structure. Using 16S rRNA gene sequencing, we show that microbial specificity of two major life stages (i.e. adults and larvae) was maintained whether corals were transplanted on algal-removed or algal-dominated bommies. While our result revealed a remarkable stability of *P. acuta* adult microbiome, parental exposure to dense macroalgal assemblages influenced the microbiome of their larvae and their subsequent performance throughout the recruitment process, thereby indicating cross-generational effects under coral–macroalgae competition. Our data provide an additional mechanism by which macroalgae perpetuate their dominance on degraded reefs. The cumulative impacts of low larval survival and low survival of the juvenile phenotypes brooded by parents exposed to macroalgae in algal-dominated conditions may dramatically disrupt coral recovery trajectories. These results further suggest that algal-induced parental versus offspring environmental effects can form complex interactions across coral development stages. Deciphering between parental and environmental drivers of offspring success with respect to coral–macroalgal competition confers promising areas to further investigate the cryptic competitive mechanisms of macroalgae. Such knowledge will be valuable for predicting benthic community changes and providing science-based guidelines for reef conservation.

## Data Availability

Metabarcoding data can be publicly accessed under the NCBI BioProject PRJNA1024906. All data and R scripts can be downloaded from the Dryad repository [74]. Supplementary material is available online [75].
